# Primary total hip arthroplasty using a short bone-conserving stem in young adult osteoporotic patients with Dorr type C femoral bone

**DOI:** 10.1186/s13018-020-01985-z

**Published:** 2021-01-07

**Authors:** Ping Zhen, Yanfeng Chang, Heng Yue, Hui Chen, Shenghu Zhou, Jun Liu, Xiaole He

**Affiliations:** 1 Department of Joint Surgery, Institute of Orthopedics, The 940th Hospital of PLA Joint Logistics Support Force, South Binhe Road, No. 333, Lanzhou City, Gansu Province 730050 PR China; 2 Department of Joint Surgery, Institute of Orthopedics, The Dingxi People’s Hospital, Anding Road, Anding District, No. 22, Dingxi City, Gansu Province 730050 PR China; 3 Department of Joint Surgery, Institute of Orthopedics, The Second Affiliated Hospital of Xi’an Jiaotong University, Xiwu Road, No. 157, Xi’an City, Shaanxi Province 730050 PR China; 4 Department of General Practice, Xijing Hospital of Air Force Military Medical University, Xi’ an, 710032 China

**Keywords:** Primary total hip arthroplasty, Cementless, Short metaphyseal fitting stem, Type C femoral bone, Osteoporosis, Young adult

## Abstract

**Background:**

Dorr type C femoral bone exhibits a wide, stovepipe-shaped femoral canal, and thin cortices in the proximal femur. Dorr C bone combined with severe osteoporosis is an important challenge in primary hip arthroplasty. In this study, we assessed the effects of short metaphyseal fitting cementless stems on preformatted primary total hip arthroplasties in young adult osteoporotic patients with this femoral presentation.

**Methods:**

A total of 42 hip arthroplasties were performed in 35 young patients (range 20 to 36 years) using a short Tri-lock bone preservation metaphyseal-fitting cementless femoral component between 2012 and 2017. The mean age at surgery of the 27 male (33 hips) and 8 female (9 hips) patients was 27.5 years (range 20.3 to 35.8 years). The mean body mass index (BMI) was 20.2 kg/m^2^ (range, 16.8–23.2 kg/m^2^). According to Dorr’s criteria, all 42 femora were classified as type C bone and all femurs suffered from severe osteoporosis (Singh index ≤ 3).

**Results:**

The mean follow-up period was 5.5 years (range 3.0 to 8.0 years). The clinical and functional results improved for the Harris hip score, WOMAC, and UCLA activity scores. The Harris Hip score improved from 48.0 ± 8.0 (range 38.0 to 61.0) preoperatively to 87.0 ± 9.0 (range 77.0 to 92.0) at 12 months after surgery and 91.0 ± 8.0 (range 85.0 to 98.0) at final follow-up. The preoperative UCLA activity score was 3.0 ± 0.5 points (range, 1.0–4.0 points), which significantly improved to 7.5 ± 0.7 points (range 6.0 to 8.0 points) at the final follow-up. No patient exhibited thigh pain at the final follow-up. The mean stem-to-canal fill percentages were 97% ± 2.1% (anteroposterior view at midstem). For stem alignment, 40 hips (95.2%) of the femoral stem were positioned neutrally to 3° of varus with reference to the femoral shaft axis. The remaining two were positioned at 4° varus to 4° valgus. Radiographic evaluation showed good osteointegration of the implants in follow-up.

**Conclusions:**

Based on the tapered-wedge design and proximal porous coating, the shortened tapered conventional stem can achieve reliable stability through neck filling and metaphyseal fixation, which does not depend on the isthmus hoop stress. This stem was suitable in severe osteoporotic patients with type C bones in young adults who presented with a correspondingly straightened femoral canal with a wide isthmus and thin cortex.

## Introduction

As described by Dorr et al. [1], type C femoral bone, characterized by wide canals, presents a challenge when choosing cementless femoral stems in patients undergoing total hip arthroplasty [2–6]. Type C bone is found predominantly in older postmenopausal women [1, 2] but is also relatively common in young adults with inflammatory joint diseases [7] such as rheumatoid arthritis (RA) and ankylosing spondylitis (AS). Patients with Dorr type C bone show a wide, stovepipe-shaped femoral canal, and thin cortices in the proximal femur [3]. The combination of an abnormal bone shape and a presumed poorer local biological environment has limited the use of conventional primary femoral prostheses in these patients [4–6]. Traditionally, femoral fixation in these patients was been achieved with polymethylmethacrylate bone cement in total hip arthroplasty (THA) [2, 5, 8]. However, the long-term performance of cemented stems may be compromised by fixation loosening, osteolysis, and a suboptimal revision setting [4, 6, 7].

For the past decade, novel techniques have been developed and more advanced formal implant designs have become available [4–6], which have prompted the use of cementless reconstruction of the femur in the presence of unusual proximal femoral anatomy [9–12]. Nevertheless, most primary cementless femoral prostheses cannot provide an excellent geometric matching to the stovepipe-shaped Dorr type C femoral canals, particularly in young adult patients with severe osteoporosis and an extremely wide isthmus [4–6]. In practice, conventional femoral prostheses cannot achieve reliable fixation and stability in abnormal femoral alterations because the wide isthmus loses metaphyseal hoop stress in cementless stems [4–11, 13].

Short bone-conserving cementless stems have been introduced to preserve proximal bone stock and allow for a more physiological proximal loading [14, 15]. With the tapered-wedge design and shorter length or reduced distal end of the conventional cementless stem [16–19], the shortened tapered stems can achieve stable fixation in the proximal femur, which does not depend on metaphyseal hoop stress in the wide isthmus. Meanwhile, decreased resection of the upper femur and/or less reaming of the femoral shaft is beneficial for preserving the femoral canal and facilitating future revision in young adults. Designed for proximal stress transfer, this approach may avoid proximal-to-distal mismatch seen with conventional designs in Dorr type A femurs [1].

In this study, we applied short Tri-lock bone preservation metaphyseal-fitting cementless femoral component to primary THA in young adult osteoporotic patients with type C bone. We believed that this type of short bone-conserving cementless stem would allow for stable biologic fixation in a wide spectrum of femoral geometries.

## Materials and methods

**Fig. 1:**
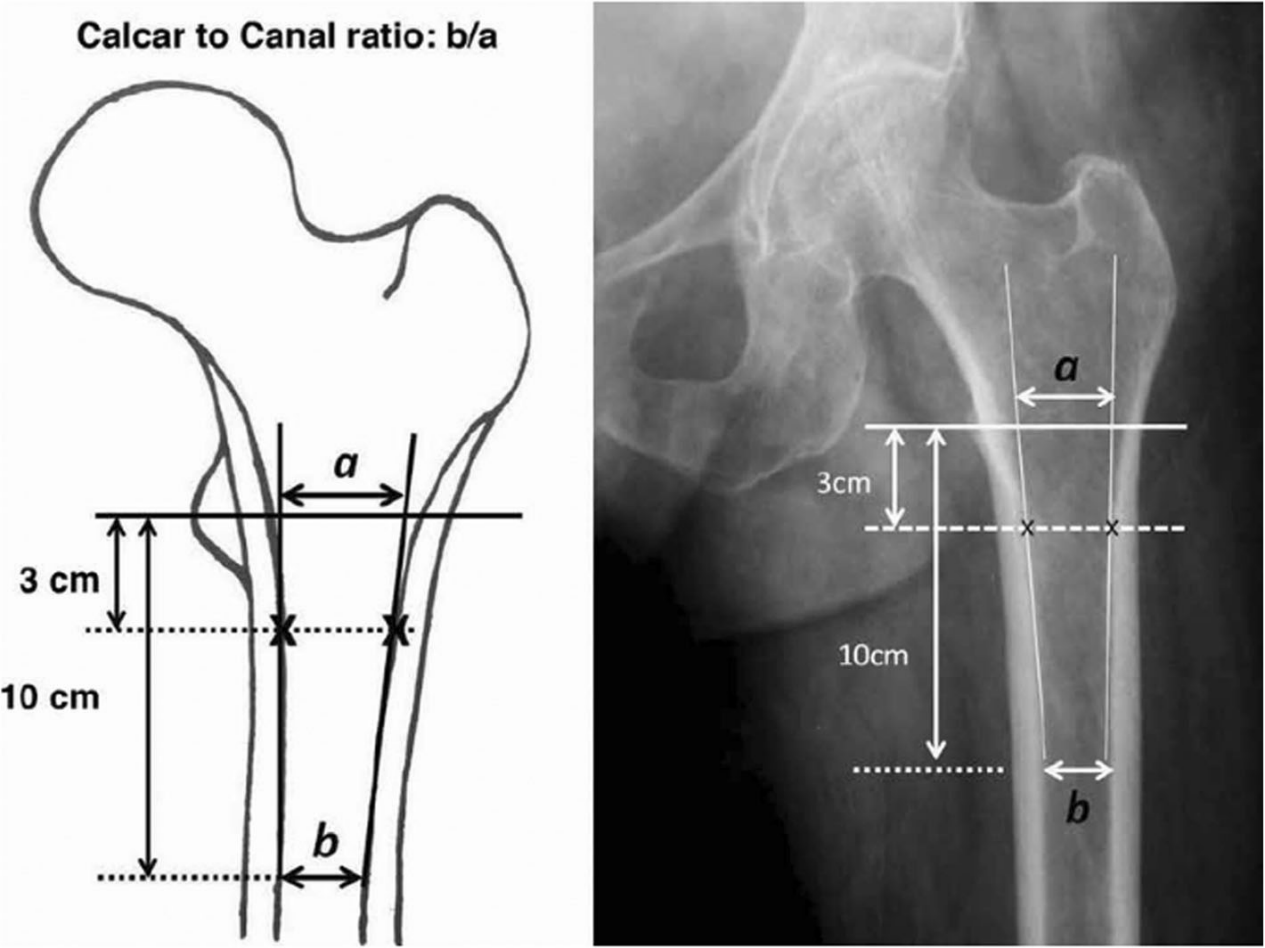
Canal-to-calcar ratio

We retrospectively assessed a consecutive series of 42 hips in 35 young adult patients with type C femoral bone of Dorr classification [1], which underwent primary THA between January 2012 and February 2017 for hip disease. The short, Tri-lock bone preservation metaphyseal-fitting cementless femoral component (Tri-Lock, DePuy, Warsaw, IN, USA) was used in every THA (Fig. 1). Patients were excluded from the study if the femoral morphology was classified into Dorr A or Dorr B, and the patients were older than 36 years or younger than 20 years from the date of operation.

There were 27 males (33 hips) and 8 females (9 hips) who underwent 35 unilateral and 7 bilateral procedures. The mean age of the patients at the time of the index arthroplasties was 27.5 years (range 20.3 to 35.8 years). The mean body mass index (BMI) was 20.2 kg/m^2^ (range 16.8 to 23.2 kg/m^2^). The hip diseases were originally rheumatoid arthritis (RA) in 20 hips (25 patients), ankylosing spondylitis (AS) in 10 hips (12 patients), and osteoarthritis in 5 hips (5 patients). All 35 patients were living independently before admission. For each patient, a complete medical history was collected. Pain and grade of disability were assessed in terms of limitation of hip range of motion and restrictions on walking daily activities. Clinical evaluation was rated with the Harris hip score [20]. For all patients, standard radiographs of the pelvis in the anteroposterior view and of the affected hip in the axial view were obtained. In radiographs, femoral bone status was evaluated before the operation [21], and the appropriate implant was chosen based on preoperative templating (see Table 1 patient demographics).

**Table 1:** Patient demographics

Demographics	Data
Gender	27 male (33 hips) and 8 female (9 hips)
Mean age	27.5 ± 3.7 years (range 20.3 to 35.8 years)
Mean height	167 ± 6.8 cm(165.5 ± 7.6 cm)
Mean weight	62.7 ± 3.6 kg (61.5 ± 7.1 kg)
BMI	20.2 kg/m^2^ (range, 16.8–23.2 kg/m^2^)
Disease	Rheumatoid arthritis (RA) in 20 hips (25 patients), ankylosing spondylitis (AS) in 10 hips (12 patients), osteoarthritis in 5 hips (5 patients)
Follow-up time	5.5 ± 1.1 years (range, 3.0–8.0 years)
Femoral cavity	0.76 ± 0.30 (range 0.67 to 0.92)
Femoral canal dimension(isthmus)	18.7 ± 2.6 mm (range 15.8 to 22.6 mm)
Femoral stem prosthesis subsidence in the last follow-up	0.39 ± 0.21 (range 0.22 to 0.47)

### Radiographic evaluation

**Fig. 2:**
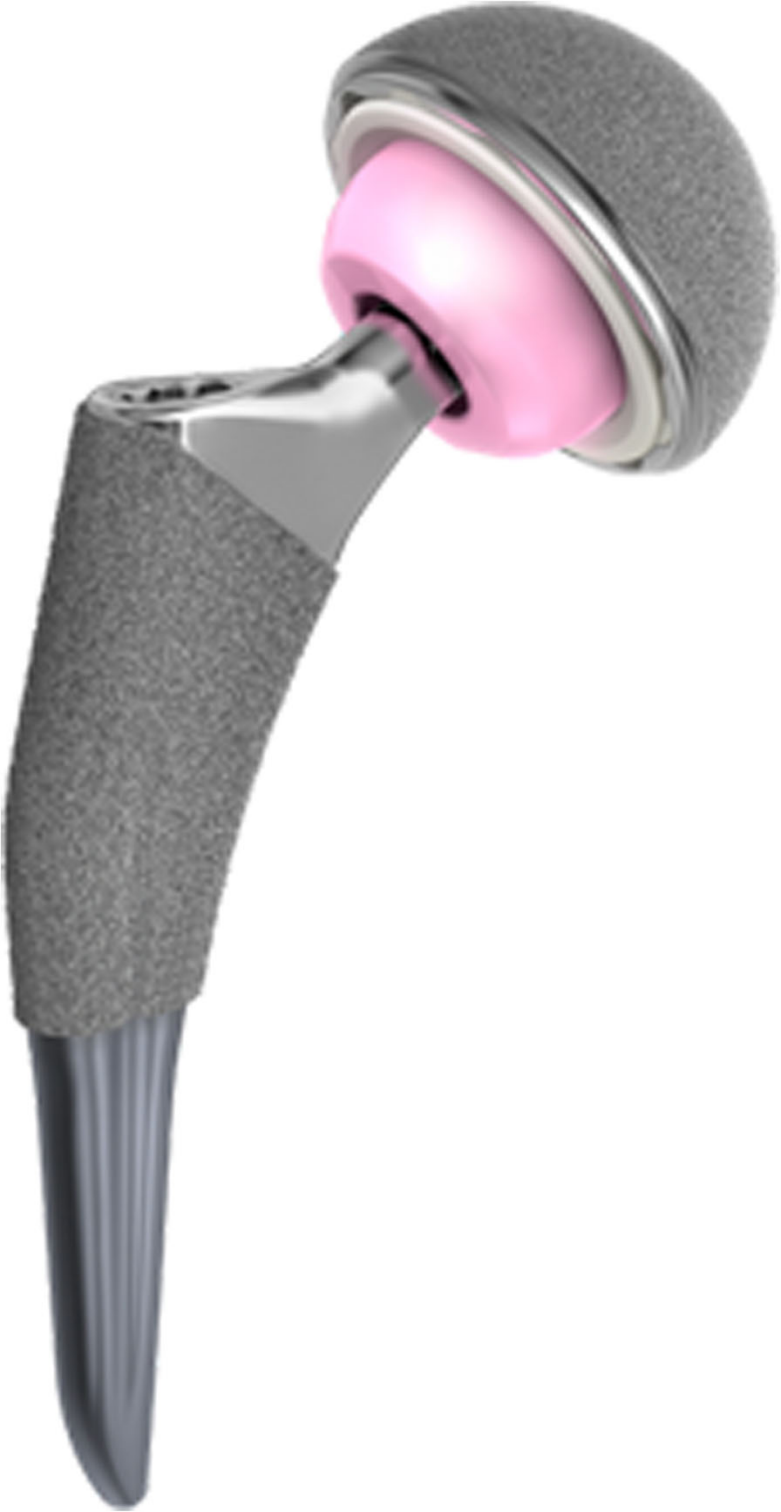
The type-4 stems are rarely neck-preserving and often extend to the upper diaphysis. With their tapered-wedge design and proximal porous coating, they achieve fixation proximally. These are similar to conventional, proximally porous-coated tapered designs with a shorter length or reduced distal end of the stem

The femoral morphology was classified according to Dorr et al. [1]. The canal-to-calcar ratio (CCR) was calculated as the fraction of the isthmus canal width divided by the calcar canal dimension. Antero-posterior hip radiographs had been taken with the X-ray beam directed toward the femoral head while the patient is supine with the foot internally rotated 15°to obtain best views of the femoral neck. X-ray tube was positioned 100 cm from focal plane of film cassette to yield an image at 20% magnification. All radiographs were taken with the same digital X-ray machine at 70 kVp and 25 mAs. CCR was calculated as the ratio of the isthmus canal width divided by the calcar canal dimension (Fig. 2) [16]. According to the Dorr classification, a CCR > 0.64 is considered to indicate type C bone [1]. The transverse diameter of the medullary canal and the thickness of the cortex of the femur were also assessed. The femoral canal dimension at the isthmus was measured on the anteroposterior view of the pelvis and the cortical index is a quotient calculated as the thickness of the cortex divided by the diameter of the femur 10 cm from the midpoint of the lesser trochanter [21]. Femoral bone morphology was classified as Dorr type C bone in all 42 hips (described as having a stovepipe shape with a wide femoral canal and thin cortices). In the anteroposterior view, the average CCR in these 42 hips (35 patients) was 0.76 ± 0.30 (range 0.67 to 0.92), the average femoral canal dimension was 18.7 ± 2.6 mm (range 15.8 to 22.6 mm) at the isthmus, and the average cortical index was 0.39 ± 0.21 (range 0.22 to 0.47). Meanwhile, all femurs suffered from severe osteoporosis (Singh index ≤ 3).

### Operative technique

THA surgeries were performed by two senior surgeons through a posterolateral approach in the lateral position. A cementless Pinnacle acetabular component (Pinnacle, DePuy, Warsaw, IN, USA) and a Tri-Lock bone preservation stem (Tri-Lock, DePuy, Warsaw, IN, USA) were used in all hips. These cup components were press-fitted after the acetabulum had been under-reamed by 2 mm. Two or three screws were used for additional fixation in 20 hips and the remainder did not require any screw fixation. We fixed the acetabular component position between 40° and 45° of inclination and between 20° and 30° of anteversion and attempted to restore hip offset according to the opposite side. For eight hips (eight patients) with deep acetabulum or protrusio acetabuli secondary to rheumatoid arthritis, hip restoration was performed using impacted autologous bone grafting combined with a hemispheric press-fit cementless acetabular cup.

All patients received a Tri-Lock cementless femoral component with a 28 mm alumina ceramic femoral head (BIOLOX1-forte; CeramTec, Plochingen, Germany) (Fig. 2). The femoral component was inserted with a press-fit technique. The proximal femur was prepared with broaches; reamers were never used. While taking care to maintain proper alignment and version, we advanced the broaches down the femoral canal. We increased the broach size until intimate contact was made between the broach and the medial and lateral cortices. The final size was achieved when the broach maintained axial and rotational stability and was at a seating level that recreated the proper leg length. Trial neck segments and trial heads were available to assess proper component position, joint stability, range-of-motion, and leg length. Standard and high-offset options were available for each stem size. We selected the stem size that corresponded to the final broach. In the area of Gription™ coating, the stem prosthesis was oversized by 0.25 mm per side relative to the broach. We introduced the implant into the femoral canal by hand while orienting the implant with proper alignment and version. Using moderate mallet blows, we advanced the stem into position. Leg length was intra-operatively determined by measuring the distance between the lesser trochanter and the centre of the femoral head. No intra-operative radiographs or image-based navigation were used. This study conformed to the Declaration of Helsinki as revised in 2008 and was authorized by the ethical committee of the author’s institution. All patients provided informed consent for this study.

The patients were allowed to stand on the second postoperative day and progress to full weight bearing with crutches as tolerated. They were advised to use a pair of crutches for 6 weeks and walk with a cane thereafter if required. All patients were able to stop using the cane in 3 months.

Clinical and radiographic follow-up was undertaken at 3 months, 1 year, and yearly thereafter. The Harris hip score [20] and the WOMAC Score [22] were determined before surgery and at each follow-up examination. Patients scored thigh pain on a 0- to 10-point visual analog scale (0 = no pain, 10 = severe pain). The level of activity of the patients after THA was assessed using the UCLA activity score [23]. We defined a limp as mild if patients moved their trunk and head 5 cm over the affected hip, moderate if they moved 5 to 10 cm, and severe if they moved more than 10 cm before the stance phase of gait.

A supine anteroposterior radiograph of the pelvis with both hips in 15° internal rotation and no abduction and a cross-table lateral radiograph were obtained immediately after operation and at each follow-up visit. The stem alignment, stem-to-canal fills, biological fixation, and subsidence were assessed in the anteroposterior and lateral planes. The radiographs were analyzed by a research associate (JL) who had no knowledge of the patient’s identity. Stem alignment in the anteroposterior radiograph was defined as varus if the tip of the stem was lateral by > 2 mm to a perpendicular line drawn down the femoral shaft and as valgus if the tip was medial to this line by > 2 mm. The stem-to-canal fill percentage [24] was assessed from the proximal-to-distal section for the anteroposterior projections. Moreover, stability of the femoral component was determined using the criteria of Engh et al. [25]. They were classified as osseointegrated, fibrous stable, or unstable [26]. Components that showed spot welds were considered osseointegrated. Those that lacked definite ingrowth but had no progressive lucency or change in position were designated as fibrous stable, and those with clear signs of loosening, including axial or angular migration, were classified as unstable. Subsidence was determined as described by Pelligrini et al. [27], and these values in the anteroposterior radiographs taken 1 week after the operation were compared to those taken at the final follow-up to define the subsidence. To estimate the leg length discrepancy radiographically, we drew a reference line across the bottom of the ischium and measured the distance from the lesser trochanter (or greater trochanter) landmark to the reference line on each side. The difference between the two represents the radiographic leg length discrepancy. Clinical examination can be used to determine the actual leg length irregularity.

Changes in Harris hip score were evaluated using a paired *t* test. WOMAC and UCLA activity scores and bone density results were evaluated using a paired t test. All statistical analyses were performed using SPSS1 version 18.0 (SPSS Inc, Chicago, IL, USA). Statistical significance was set at p values of *P* < 0.05.

## Results

The mean follow-up was 5.5 years (range 3.0 to 8.0 years). The clinical and functional results improved for all scores. At final follow-up, 33 patients (94%) had no detectable limp and 2 (6%) had a mild limp that was related to leg length discrepancy and weakness in the abductor muscle. The ability to use stairs and public transportation, put on footwear, and cut toenails improved after the operation. The Harris hip score improved from 48.0 ± 8.0 (range 38.0 to 61.0) preoperatively to 87.0 ± 9.0 (range 77.0 to 92.0) at 12 months after surgery and 91.0 ± 8.0 (range 85.0 to 98.0) at final follow-up. The preoperative UCLA activity score was 3.0 ± 0.5 points (range, 1.0–4.0 points), which significantly improved to 7.5 ± 0.7 points (range 6.0 to 8.0 points) at the final follow-up, which was significant (*p* = 0.001). No patient had thigh pain at the final follow-up (see Table 2 comparisons of hip joint functions).

**Table 2:** Comparisons of hip joint functions in the 35 patients receiving THA before and after the operation (means ± *s*, point)

Time of assessment	Pain	Function	UCLA activity score	Range of motion	Harris hip score	Hip range of motion (°)
Preoperatively	18.5 ± 4.8	15.8 ± 3.9	3.0 ± 0.5	1.6 ± 0.8	48.0 ± 8.0	45.8° ± 8.5°
Last follow-up	10.2 ± 2.6	42.1 ± 5.5	7.5 ± 0.7	4.3 ± 0.9	87.0 ± 9.0	108. 5° ± 12.0°
*t* value	30.417	142.601	113.112	217.681	230.727	144.43
*P* value	< 0.001	< 0.001	< 0.001	< 0.001	< 0.001	< 0.001

Femoral stem filling was assessed as a percentage at the level of just below the lesser trochanter based on postoperative AP X-rays, which was calculated as SW (stem width)/ID (inner diameter of the femur) × 100 in millimeters. The mean stem-to-canal fill percentages were 97% ± 2.1% (at midstem, anteroposterior view). In stem alignment, 40 hips (95.2%) of the femoral stem were positioned neutral to 3° of varus with reference to the femoral shaft axis. The remaining two were positioned at 4° varus to 4° valgus. Accurate reconstruction of leg length was observed in this group. Leg length was similar with a mean of 0.7 mm (0.3–1.5 mm) in 20 hips. Leg length was within the range of – 2 mm (short) to 2 mm (long) in 13 hips. Leg length discrepancy was 4 mm lengthened in two cases and no limb lengthening of more than 5 mm was observed.

**Fig. 3:**
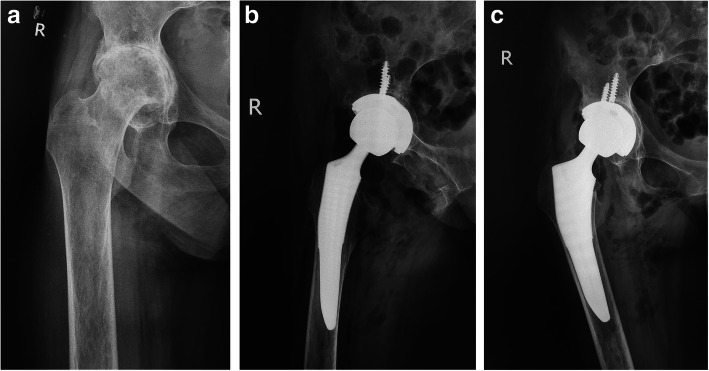
Radiographs illustrate the case of a 36-year-old female patient who had rheumatoid arthritis (RA) in the right hip. **a** An AP view of the hip shows a Dorr C femoral bone combined with severe osteoporosis. Note the deep acetabulum and extremely widened femoral canal with very thin cortices. These parameters are limited by the use of most primary conventional prosthesis or hip resurfacing arthroplasty. **b** Total hip arthroplasty (featuring placement of a cementless porous acetabular component and a short Tri-Lock bone preservation stem, Tri-Lock, DePuy) was accompanied by acetabular reconstruction with an autologous bone graft. An AP X-ray taken after surgery revealed that the initial stability of the acetabular cup and stem was good. The radiolucent line between the acetabular wall and peripheries of the cup is bone grafting by autologous femoral head impaction. **c** Lateral view postoperatively

**Fig. 4:**
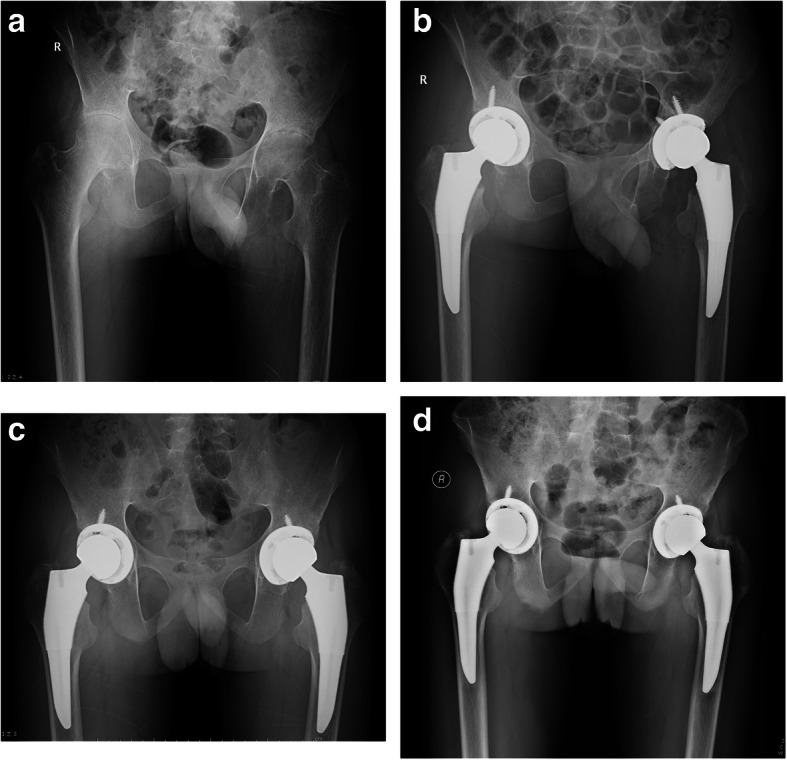
Radiographs illustrate the case of a 21-year-old male patient who had bilateral ankylosing spondylitis (AS). **a** An AP view of both hips before surgery shows the narrowed hip joint spaces, damaged femoral head, and Dorr type C femoral bones. **b** A pelvic X-ray taken immediately after surgery showed that the initial stability of the acetabular cup and short stem were achieved by press fit and good canal filling of the short stem. **c** An AP view of both hips taken 1 year postoperatively reveals that the acetabular cup and stems are well fixed in a good position in both hips. Grade 2 calcar resorption is evident in both hips, but periprosthetic bone stock is preserved without stress shielding-related bone osteopenia. **d** An AP view of both hips taken 8 years postoperatively demonstrates that the femoral components are in a satisfactory position without osteolysis and subsidence. Note the radiographic signs of calcar rounding and the increase in bone density in this area. No component migrated superiorly or medially

Osseointegration was seen in all hips. Based on the Engh classification [25], 42 hips showed radiographic evidence of bone ingrown prosthesis. At the final follow-up (average 5.5 ± 1.1 years), 40 hips (95%) showed grade 1 stress shielding in the calcar region. No hip exhibited grade 3 stress shielding, no hip had a subsidence of more than 1.0 mm, and no acetabular or femoral osteolysis was identified. Cortical hypertrophy of the femur was seen in 18 hips. This hypertrophy was most common in Gruen zones 3 and 5 (Figs. 3a–c and 4a–d).

During the follow-up period, no adverse events such as infection, deep venous thrombosis, loosening, or periprosthetic fracture were recorded in the study group.

## Discussion

The morphology of the proximal femur differs according to age, race, sex, and lifestyle [2, 3, 28]. Some pathologic factors may also affect the geometry of the femur, such as rheumatoid arthritis, atrophic osteoarthrosis, osteoporosis, and some metabolic bone diseases [7, 29, 30]. Type C bone is found predominantly in women of older age and with lower body weight [1] and it has both structural and cellular compromise. The cortices are thin with correlated loss of the medial and posterior cortices resulting in a “stovepipe” shape of the intramedullary canal [3]. The significant decrease in cortical indices and the increase in CCRs reflect these structural changes. These structural changes, in combination with cellular abnormalities, create a less favorable environment for implant fixation. These changes also complicate joint replacement and may negatively affect the immediate fixation and long-term survival of the prosthetic implant [1, 3–6, 31]. Dorr C bone also poses challenges for the insertion of cementless stems because type C bones often present a straightened femoral canal with a wide isthmus and thin cortex [3, 30]. Most primary conventional cementless stem implants cannot provide a close geometric matching to the stovepipe canals with a wide isthmus [2–6, 13, 32], and the enlarged isthmus can lose its metaphyseal hoop stress for the most primary conventional cementless taper stems [2]. A sufficiently tight fill and fit cannot be achieved using even the largest femoral prosthesis, which can lead to stem slippage or excessive motion that prevents stem ingrowth. Cementless revision femoral prosthesis has been applied to achieve endosteal stem fit and fill the primary THA in type C femoral bones [13]. However, there are some concerns that more stress shielding may occur [33] because of the large stem placed in the proximal femur. In addition, more erosion of the femoral inner canal can occur by placing a large length and diameter stem in an overall weakened bone, which could decrease the reserve of the bone stock.

The Dorr C stovepipe femur can be seen with severe osteoporosis because some conditions can create osteoporotic bones. In practice, patients requiring long-term steroid use from organ transplantation or rheumatological conditions may simultaneously develop osteonecrosis and osteoporosis [33]. Type C femoral bone combined with severe osteoporosis is becoming a common challenge faced by surgeons performing primary hip arthroplasty. Besides the poor bone quality with a more enlarged femoral canal complicating stem matching and fixation, surgery is also associated with a range of adverse outcomes, such as intraoperative fracture, periprosthetic osteolysis with implant migration, and postoperative periprosthetic fracture [33, 34]. Thus, these shape and structural changes result in the preferred use of cemented implants in Dorr C osteoporotic bone [4, 6, 35] because some mismatches can be accommodated using the avoid-filling capacity of the cement layer in a cemented hip system [3]. The only limitation is when the femoral canal becomes so wide that even the largest cemented stems begin to have a very thick cement mantle [13]. Recommended mantles are 1–2 mm, and severely undersized femoral components may show early loosening [36, 37]. Even cementation becomes difficult in these severe Dorr C osteoporotic bones with profoundly wide and thin cortices of femur [37]. Furthermore, fixation loosening of the cemented femoral component has remained the leading revision problem, particularly in young patients [38]. Meanwhile, fat embolism, pulmonary microemboli, and cardiac arrest associated with cementing are still potential risks [39, 40] because a large amount of cement is required to fill the widened canal in the enlarged femoral canal.

To address this difficult issue, a case series was performed with many other choices of hip arthroplasty or other types of femur stems to address Dorr C osteoporotic bone in young adults [32, 33]. Total hip resurfacing arthroplasty is a good alternative to conventional total hip arthroplasty and can provide excellent results in terms of implant survivorship after careful patient selection [41, 42]. However, the poor bone mineral density may contribute to increased fractures of the femoral neck and head or pathological fractures in patients with severe osteoporosis, particularly in patients comorbid with rheumatologic disorders [43, 44]. Osteopenia or weakness of the femoral head may lead to femoral component loosening because it is mechanically unfavorable to have resistance on the femoral head and neck by stresses transmitted through the implant. Meanwhile, hip resurfacing arthroplasty is not indicated in young women who wish to become pregnant in the future as pregnancy-related complications and adverse effects of metal on metal debris on the fetus are unknown [42].

With current advances in prosthetic designs, there is growing interest in bone-conserving short stems to preserve proximal bone stock and provide physiologic loading to the proximal part of the femur [12, 15]. The short, metaphyseal fitting cementless femoral stem was designed to require less resection of the upper femur and/or less reaming of the femoral shaft [45, 46]. This serves a dual purpose of facilitating future revision while providing a postoperative state closely mimicking the original functioning hip. Preservation of the femoral neck provides greater torsional stability and reduces distal migration of the femoral stem [47, 48]. The absence of any diaphyseal fixation can be used to achieve proximal load transfer to reduce stress shielding and ignore the type of femoral canal. It also preserves the femoral canal and femoral elasticity, allowing for easy revision.

The short bone-conserving cementless stems have been introduced in young patients with type C bone [49]. The short stem is suitable for most femur types because the initial stability of the stem can be achieved by neck filling and metaphyseal fixation, which does not depend on isthmus hoop stress compared to conventional primary femoral prostheses. In fact, most primary conventional implants cannot provide a close geometric matching to the extremely stovepipe canals [2–6, 13, 32]. We used the type-4 stem [12] in primary total hip arthroplasty in young adult osteoporotic patients with type C bones. The type-4 stem is a shortened conventional design with primary fixation in the proximal femoral metaphysis. As opposed to other types of short bone-conserving stems [12], type-4 stems are rarely neck-preserving and often extend to the upper diaphysis. With their tapered-wedge design, they achieve fixation in the proximal femur. These are similar to conventional, proximally porous-coated tapered designs with a shorter length or reduced distal end of the stem [17–20]. The “fit and fill” of the stem can be achieved by impaction of cancellous bone in the metaphyseal region with a larger proximal geometry. In our practice, type-4 stems are suitable for severe osteoporotic young patients with type C bones who present with a correspondingly straightened femoral canal profiled with a wide isthmus and thin cortex. These changes increase the difficultly of performing replacement using other types of hip arthroplasty or other conventional femoral stems with a stovepipe canal combined with severe osteoporosis [17]. There are several proposed advantages of this type of short stem, including easier insertion through smaller incisions and less invasive techniques [20], simpler femoral preparation with a “broach-only” system, and the basic inherent bone conserving nature allowing for more favorable conditions in the potential revision setting.

This study had certain limitations. First, it was retrospective in nature and included a relatively small series of patients. Second, the duration of follow-up was short and insufficient to allow conclusions to be drawn because our prostheses only showed good results at 3 to 8 years (mean, 5.5 ± 1.1 years) after the operation. Third, our migration analyses of the stem did not use more precise methods such as roentgen stereophotogrammetry (rSA) or Ein-Bild-roentgen-femoral component analyses [18, 50]. Meanwhile, we did not use dual-energy X-ray absorptiometry to study bone mineral density changes around the femoral stem, which is considered the most reliable tool for evaluating bone remodeling after THA using different stem designs and is more sensitive and precise than conventional X-ray. Fourth, bone remodeling around the short stem was not observed comprehensively in long-term follow-up. Finally, we did not perform interobserver variability studies of the radiographic results to confirm the measurements by the single observer, which may lead to bias in interpreting the radiographs, leading to underestimation or overestimation.

## Conclusions

In summary, the short, metaphyseal-fitting cementless femoral component is similar to conventional, proximally porous-coated tapered designs with a shorter length or reduced distal end of the stem. It provides stable fixation without the need for diaphyseal fixation in severe osteoporotic young patients with type C bone. We believe that the lateral flare of the stem provides axial and torsional stability, which provides a more natural loading of the proximal femur. Further long-term follow-up is required to confirm these short-term results.

## Data Availability

The data and materials are available from the medical records department of the 940th Hospital of PLA, the Second Affiliated Hospital of Xi’an Jiaotong University and Xijing Hospital. The datasets used and analyzed during the current study are available from the corresponding author on reasonable request.

## Abbreviations

THA: Total hip arthroplasty
Tri-Lock BPS: Tri-Lock bone preservation stem prosthesis
